# Evaluation of Astringent Compounds Using Electronic Tongue Technology

**DOI:** 10.1002/fsn3.71276

**Published:** 2025-11-26

**Authors:** Meng‐Yao Wang, Zhao‐Lin Sun, Juan Lü, Hao Zhu, Zeng‐Hui Zhang, Xi Zhang, Zhi‐Gang Guo, Yao Wang, Jing Yang

**Affiliations:** ^1^ College of Tobacco Science and Engineering Zhengzhou University of Light Industry Zhengzhou China; ^2^ Technical Center of Shaanxi Tobacco Industry Co. Ltd. Xi'an China

**Keywords:** astringent, cluster analysis, electronic tongue, principal component analysis, sensory evaluation

## Abstract

The electronic tongue (E‐tongue) is an emerging technology that enables the rapid and objective evaluation of various astringent compounds. This study investigated E‐tongue measurements and human sensory evaluation to analyze the concentration‐dependent astringency responses of seven compounds. Difference analysis, principal component analysis (PCA), and cluster analysis were applied to differentiate the compounds based on their taste profiles. The results revealed distinct relationships between E‐tongue response values and concentration. Specifically, epigallocatechin gallate (EGCG) and epigallocatechin (EGC) exhibited positive correlations with concentration, whereas tea polyphenols, tannic acid, and procyanidin showed negative correlations. In contrast, gallic acid and chlorogenic acid produced weak astringency responses. Difference analysis demonstrated significant taste variations across concentrations; PCA and cluster analysis further validated the E‐tongue's capability for distinct discrimination of astringent compounds. Regression analysis between E‐tongue measurements and human sensory scores demonstrated strong correlations for EGCG, EGC, tea polyphenols, tannic acid, and procyanidin, with procyanidin and EGCG exhibiting the best linearity. Predictive models achieved high accuracy (*R* ^2^ > 0.9, RMSE < 10%) in validation, demonstrating the E‐tongue's reliability as an alternative to sensory evaluation for astringency assessment.

## Graphical abstract

This study investigated the capability of the electronic tongue to evaluate diverse astringent compounds. The integrated predictive model combining sensory evaluation and E‐tongue data accurately quantified astringency intensity, while multivariate analysis enabled clear discrimination and classification among different types of astringent compounds by the electronic tongue.

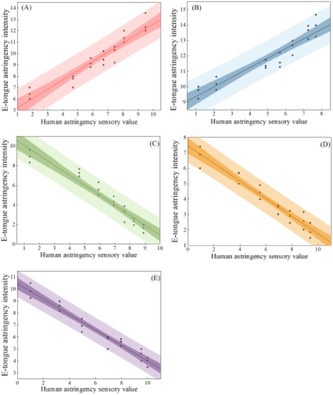

## 1 Introduction

Astringency constitutes a unique sensory phenomenon distinct from fundamental taste modalities such as acidity, sweetness, and bitterness (Jiang et al. 2014), which arise from a combination of tactile and chemical interactions rather than being purely taste‐driven. The American Society for Testing and Materials (ASTM) defines astringency as “a complex sensation involving the contraction and wrinkling of the epithelial surface, primarily induced by tannins or alums” (Zhu et al. 2025). This sensation is commonly encountered in foods such as tea, red wine, and unripe fruits. Astringent substances in food primarily originate from plant secondary metabolites (Wei et al. 2021). In tea, catechins have been identified as a major class of astringent compounds. Based on their chemical structures, catechins can be categorized into ester‐type (e.g., epigallocatechin gallate, EGCG) and non‐ester‐type (e.g., epigallocatechin, EGC) (Ye et al. 2022). A previous study reported that tea polyphenols content exhibits a strong positive correlation with perceived astringency (Wan et al. 2021). In addition, astringency in fruits has been significantly linked to polyphenols such as catechins, procyanidin, chlorogenic acid, and tannic acid (Guilois‐Dubois et al. 2021; Wang et al. 2023; Wu et al. 2022). Tannic acid dominates as the astringent compound in wine (Wang et al. 2022), persimmons (Soares et al. 2020), and grapes (Ahmad et al. 2023). These astringent compounds not only elicit oral sensory responses but also demonstrate diverse physiological benefits, including antioxidant, hypoglycemic, hypolipidemic, anticancer, and immunomodulatory effects (Lee et al. 2024; Shen et al. 2024; Wang et al. 2024), which highlight their broad potential for applications in food, pharmaceuticals, and related fields.

Current methods for assessing astringency intensity, primarily involving three methodologies: human sensory evaluation (Weber et al. 2023), polyacrylamide gel electrophoresis (PAGE) (Wang, Wang, et al. 2021), and chemical precipitation assays (Pavez et al. 2022), are limited by subjectivity, poor reproducibility, operational instability, and complex procedures. In contrast, E‐tongue technology provides a robust alternative by simulating human taste perception through cross‐selective sensor arrays coupled with multivariate pattern recognition algorithms. This approach enables rapid, objective, and digitized quantification of key taste attributes, including acidity, astringency, and umami, with high reproducibility (Wang, Zhuang, et al. 2021). Its rapid and objective quantification of taste intensity has led to widespread applications in assessing taste quality across various food products, including beer (Nery and Kubota 2016), cheese (Valente et al. 2018), tea beverages (Zan et al. 2025), and red wine (Costa et al. 2015). Given these advantages, electronic tongue technology shows significant potential for advancing food taste quality assessment across diverse applications.

While previous studies have shown the E‐tongue can assess astringency in specific foods like tea or persimmons (Xue et al. 2017; Zhang et al. 2025), research in this area remains relatively scarce. Most studies focus on one type of food, leaving a gap in understanding how the technology performs with pure astringent compounds across different categories. This study evaluated an E‐tongue's performance in detecting various astringent compounds across multiple food matrices to establish a sensory‐prediction model, assess discriminative capacity, and develop a standardized method for rapid astringency assessment.

## 2 Materials and Methods

### 2.1 Materials and Reagents

Tea polyphenols, procyanidin, tannic acid, gallic acid (purity ≥ 98%; Zhongchen Biotechnology Co. Ltd., Henan, China), chlorogenic acid (purity ≥ 98%; Tianxingjian Biotechnology and Chemical Technology Co. Ltd., Shaanxi, China), epigallocatechin gallate, epigallocatechin (purity ≥ 98%; Xubo Technology Co. Ltd., Beijing, China). All of the above are of food grade. Internal solution, reference solution, anion solution, and cation solution, Insent Co. (Tokyo, Japan).

### 2.2 Preparation of Astringency Sample

A pilot experiment employing the three‐alternative forced choice (3‐AFC) method was conducted with 10 trained sensory evaluators. Following the protocol detailed in Section 2.4.2, we determined the effective concentration ranges for seven astringent compounds, covering the full sensory spectrum from “non‐perceptible” to “intensely astringent.” The “non‐perceivable” threshold was defined as the concentration (i.e., the recognition threshold) at which over 50% of evaluators could not distinguish the sample from the deionized water control (Pavez et al. 2022). The ‘intensely astringent’ threshold was defined as the concentration at which more than 50% of evaluators rated its astringency intensity as equivalent to 2 g/L zinc lactate, corresponding to an extremely strong sensation. Concentration points were selected based on established studies of similar systems (Soares et al. 2020; Zan et al. 2025). Seven gradient concentrations were established for model construction, with two additional levels included for validation, as detailed in Table 1.

**TABLE 1:** Concentration gradients of seven astringent compounds.

No.	Astringent	Gradient concentration (g/L)							Validation concentration (g/L)	
1	EGCG	0.15	0.50	0.80	1.00	1.20	1.60	2.40	0.6	1.4
2	Tea polyphenols	0.10	0.40	0.80	1.00	1.20	1.80	2.60	0.5	1.5
3	Tannic acid	0.10	0.30	0.70	1.00	1.20	1.60	2.40	0.5	1.5
4	EGC	0.05	0.10	0.50	0.80	1.00	1.40	1.80	0.3	1.2
5	Procyanidin	0.05	0.20	0.40	0.90	1.40	2.00	2.60	0.5	1.5
6	Chlorogenic acid	0.20	0.50	1.00	1.20	1.60	2.40	3.00		
7	Gallic acid	0.10	0.60	1.00	1.20	1.80	2.20	2.60		

Abbreviations: EGC, epigallocatechin; EGCG, epigallocatechin gallate.

### 2.3 Sensor Activation and E‐Tongue Measurement

The SA402B E‐tongue system (Tokyo, Japan) was employed for sample analysis. This instrument comprises five working sensors (AAE, CT0, CA0, AE1, C00) configured for umami, saltiness, sourness, astringency, and bitterness detection, along with two reference electrodes. Before measurement, all sensors were prepared as follows: working sensors were disassembled to replenish the internal solution, reactivated in reference solution for 24 h, then calibrated in 3.33 mol/L KCl solution; reference electrodes underwent identical hydration treatment.

During operation, sensors were zeroed in reference solution for 30 s before sample measurement. Each 30 s measurement cycle was followed by a 3 s reference solution rinse, with five replicates performed per sample. The final three stable readings were adopted for analysis. Sensor potentials were calculated using: *V* = *V*_s_ − *V*_r_, where *V* represents the normalized taste index, *V*_s_ denotes the stable potential in the sample solution (30 s average), and *V*_r_ indicates the reference solution potential (30 s average).

### 2.4 Sensory Panel

#### 2.4.1 Sensory Group Training

The study utilized seven food‐grade astringent compounds that met international safety standards. Following ISO 8586:2012 (Assessors for sensory analysis—part 1: Guide to the selection, training, and monitoring of select assessors) guidelines for sensory panel selection, 20 candidates were recruited from the university population for preliminary screening. Five basic taste solutions were prepared using reagent‐grade chemicals: quinine (bitterness), zinc lactate (astringency), sucrose (sweetness), citric acid (sourness), and monosodium glutamate (umami). Candidates demonstrating adequate taste discrimination ability were selected for subsequent training.

#### 2.4.2 Sensory Evaluation

Trained panelists were individually isolated in standardized sensory booths under controlled environmental conditions (25°C). A series of zinc lactate reference solutions, calibrated to five distinct astringency levels (Table 2), served as training stimuli. Panelists unable to consistently differentiate astringency intensities were excluded from further participation. The final panel comprised seven qualified individuals meeting all selection criteria. During evaluation, the sample presentation order was randomized. Panelists retained samples in their mouths for approximately 20 s before expectoration and scoring. The mean astringency score from all panelists was recorded as the sample's astringency value. To minimize carryover effects, a 20‐min interval was enforced between sample evaluations. Additionally, all samples were maintained at 25°C to eliminate temperature‐induced sensory variation.

**TABLE 2:** Concentration and astringency criteria for zinc lactate solutions.

Zinc lactate concentration (g/L)	Astringent score	Astringent description
0.1	0–2	Not astringent or slightly astringent
0.6	2–4	Low astringent
1.0	4–6	Moderately astringent
1.5	6–8	High astringent
2.0	8–10	Extremely astringent

### 2.5 Establishment and Evaluation of a Prediction Model

Regression analysis was performed using Origin Pro 2021 (Origin Lab Corp., Northampton, USA). Artificial sensory intensity was set as the independent variable, and E‐tongue astringency intensity was set as the dependent variable. A predictive regression model between the electronic tongue astringency values and the sensory astringency values was established for the five compounds EGCG, tannic acid, tea polyphenols, EGC, and procyanidin—using the least squares fitting method. Gallic acid and chlorogenic acid were excluded from the model due to their weak sensor responses, which rendered them unsuitable for multivariate analysis using the least squares fitting method. The 95% confidence interval (95% CI) and 95% prediction interval were calculated and plotted to evaluate the model's uncertainty and the probable range of individual predicted values. The model's predictive performance was assessed using the validation concentrations, with the coefficient of determination (*R* ^2^), root mean square error (RMSE), and mean relative error (MRE) serving as evaluation metrics.

### 2.6 Statistical Analysis

Descriptive statistical analysis was performed using IBM SPSS Statistics 26.0 (Stanford, CA, USA). Data are presented as mean ± standard deviation (SD). Significant differences (*p* < 0.05) among means are denoted by different superscript letters. For datasets meeting parametric assumptions, differences in taste indicators among compounds at the same concentration were compared using one‐way analysis of variance (ANOVA), followed by Duncan's new multiple range test for post hoc comparisons. For datasets exhibiting heteroscedasticity, the nonparametric Kruskal–Wallis *H* test was conducted, followed by a post hoc Dunn's test with Bonferroni correction for multiple comparisons.

PCA and cluster analysis were performed using IBM SPSS Statistics 26.0 (Stanford, CA, USA). PCA was performed on six primary taste indicators and two aftertaste indicators, with principal components extracted based on eigenvalues > 1; cluster analysis is the clustering of selecting the connection between groups. Graphs were produced using Origin Pro 2021 (Origin Lab Inc).

## 3 Results and Discussion

### 3.1 Response Patterns of E‐Tongue to Astringent Compounds at Varying Concentrations

**FIGURE 1:**
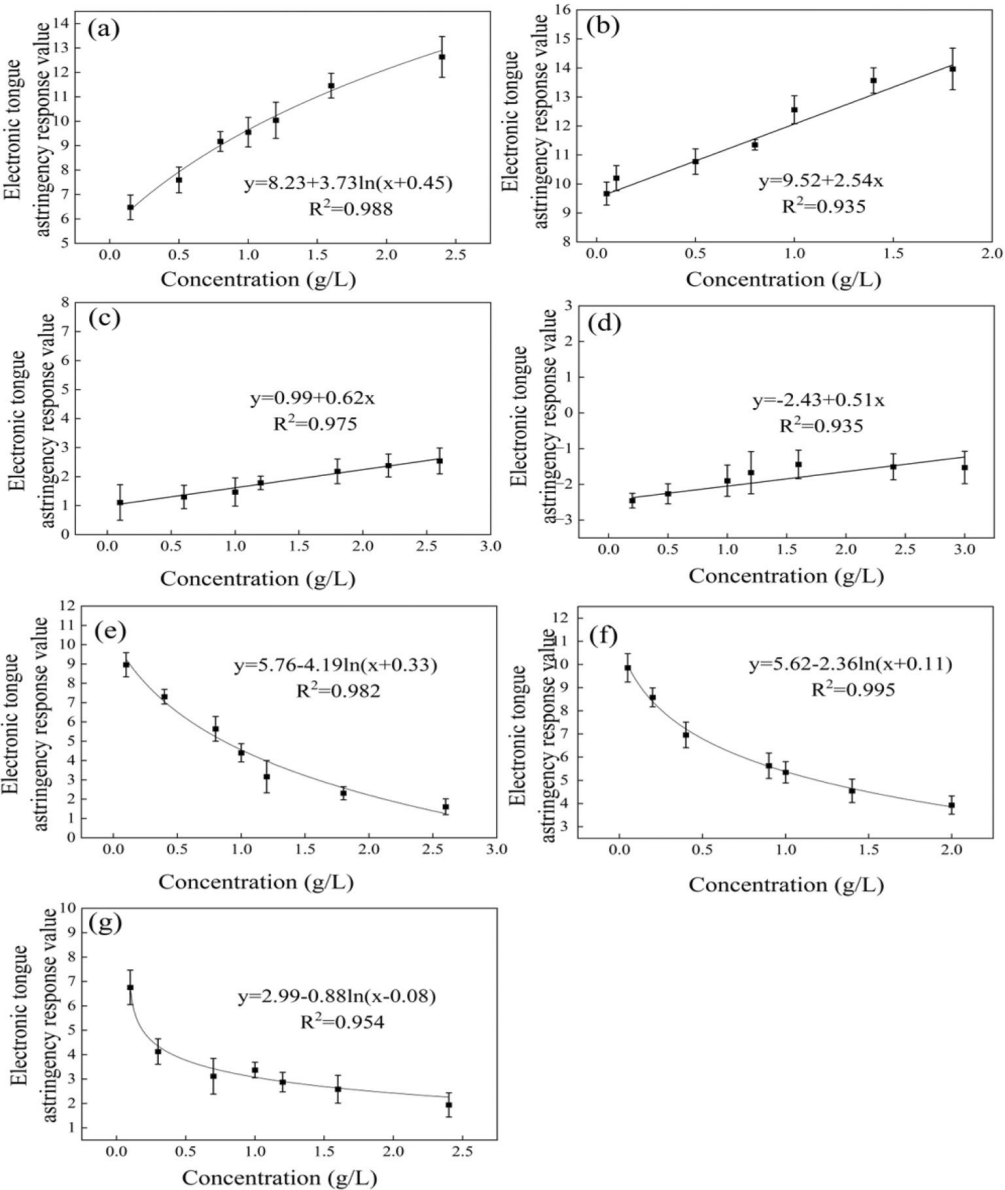
Concentration–astringency relationship of E‐tongue for different compounds taste intensity. (a) Epigallocatechin gallate (EGCG), (b) epigallocatechin (EGC), (c) gallic acid, (d) chlorogenic acid, (e) tea polyphenols, (f) procyanidin, and (g) tannic acid. The data points represent the mean ± SEM (*n* = 3).

The concentration–astringency response relationships of the seven astringent compounds (EGCG, chlorogenic acid, tea polyphenols, tannic acid, gallic acid, EGC, and procyanidin) obtained using the E‐tongue are presented in Figure 1.

The results demonstrate distinct concentration–response patterns: EGCG and EGC exhibited monotonically increasing astringency intensity with rising concentration, whereas tea polyphenols, tannic acid, and procyanidin showed a negative correlation between E‐tongue astringency intensity and concentration. Chlorogenic acid and gallic acid displayed minimal concentration‐response variation, maintaining consistently low astringency values (Figure 1). A similar correlation pattern was previously observed for the four basic taste compounds: sour, sweet, bitter, and salty. Specifically, the taste intensity measured by the E‐tongue demonstrated a positive correlation with the concentration of sour and salty substances, but a negative correlation with that of sweet and bitter substances (Tian et al. 2015). Regression analysis revealed strong logarithmic correlations (*R* ^2^ > 0.935) between concentration and astringency intensity for EGCG, tannic acid, tea polyphenols, EGC, and procyanidin, with gallic acid and chlorogenic acid following a linear relationship. This demonstrates the E‐tongue's capability to characterize fundamental concentration–astringency relationships.

Notably, sensor responses varied by compound structure. Chlorogenic acid yielded consistently negative values across all seven tested concentrations, ranging from −2.36 ± 0.23 to −1.53 ± 0.48 (Figure 1). This finding is consistent with observations reported by Liu et al. (2022) in their study on alum astringency, which also recorded negative yet concentration‐dependent increases in electronic tongue astringency responses. This phenomenon relates to the AE1 sensor array's molecular recognition mechanism (Immohra and Pein‐Hackelbusch 2017). The lipid‐polymer membrane electrodes detect flavor compounds through interfacial potential changes. Astringent compounds interacting with membrane phosphate groups may generate negative surface charges, explaining the observed negative potentials for certain analytes. As a consequence, in this single‐astringency flavor study, the astringency sensor exhibited distinct response patterns to each compound, demonstrating its ability to differentiate between different astringent stimuli.

### 3.2 Concentration–Intensity Relationship of Astringent Compounds by Human Sensory Evaluation

**FIGURE 2:**
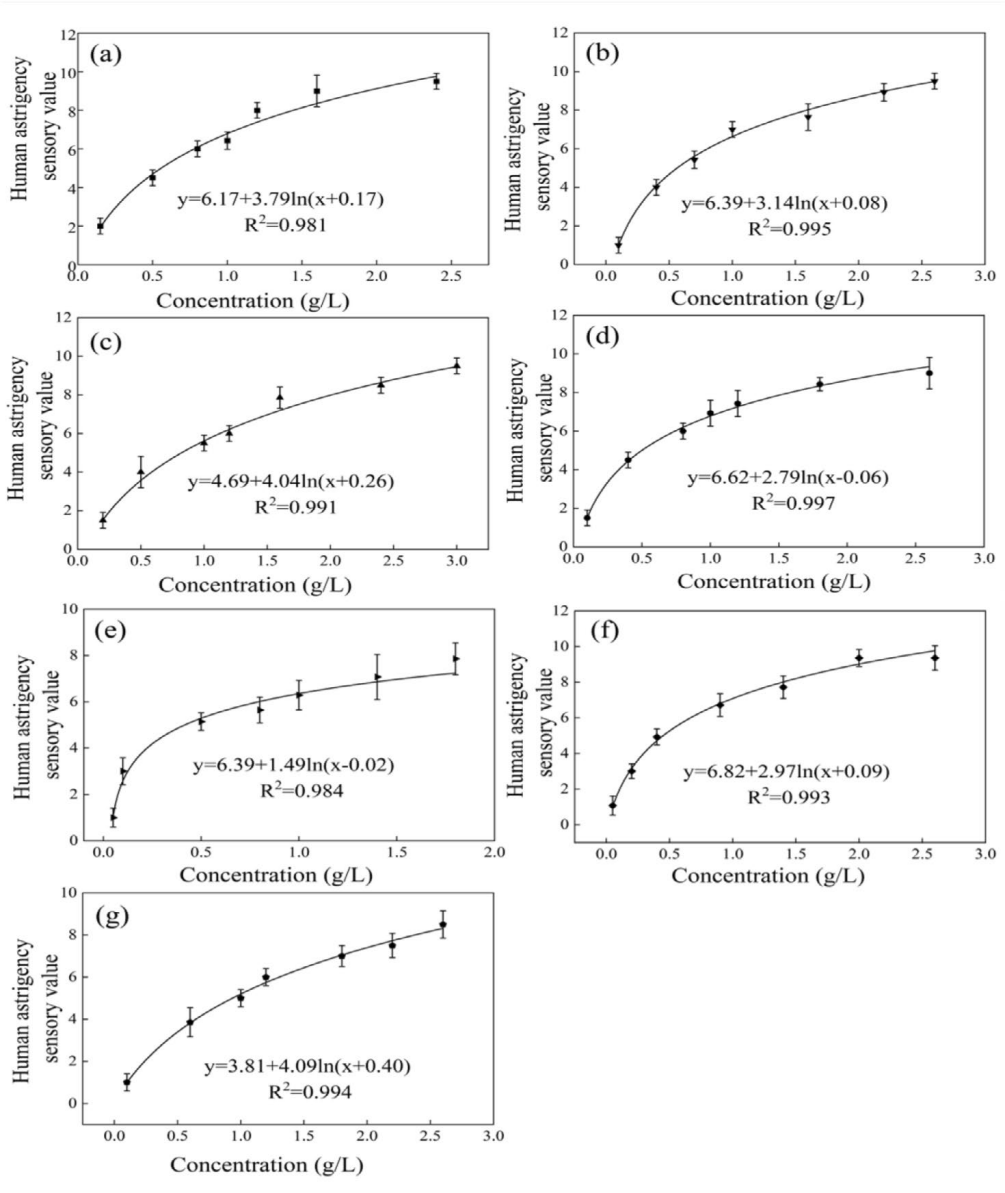
Concentration–intensity relationship of human sensory astringency for different compounds. (a) Epigallocatechin gallate (EGCG), (b) tannic acid, (c) chlorogenic acid, (d) tea polyphenols, (e) epigallocatechin (EGC), (f) procyanidin, and (g) gallic acid. The data points represent the mean ± SEM (*n* = 7).

The concentration–astringency relationships of seven key astringent compounds were systematically characterized through human sensory evaluation. All compounds exhibited concentration‐dependent increases in perceived astringency intensity, with particularly rapid escalation below 1.5 g/L followed by gradual saturation at higher concentrations (Figure 2). The slopes of the fitting curves for the seven compounds ranged from 1.49 to 4.09. Gallic acid and chlorogenic acid exhibited the largest slopes with 4.09 and 4.04, indicating that their perceived astringency was highly sensitive to concentration changes. In contrast, EGC showed the smallest slope with 1.49, suggesting a much weaker concentration dependence for this compound (Figure 2). Quantitative analysis demonstrated an excellent logarithmic fit for these relationships (*R* ^2^ > 0.982, Figure 2), demonstrating strong predictive power of the fitting equations for estimating human astringency perception. The fit was particularly accurate for tea polyphenols (*R* ^2^ = 0.997), whose logarithmic model showed the smallest deviation from the data points, indicating it was the most precise in describing the concentration–astringency relationship. The close logarithmic agreement is consistent with the Weber‐Fechner law (Wang et al. 2025), which posits that perceived intensity scales logarithmically with stimulus magnitude, thereby providing robust support for the sensory evaluation model.

### 3.3 Construction and Validation of a Quantitative Prediction Model for Astringent Compounds Using an E‐Tongue

**FIGURE 3:**
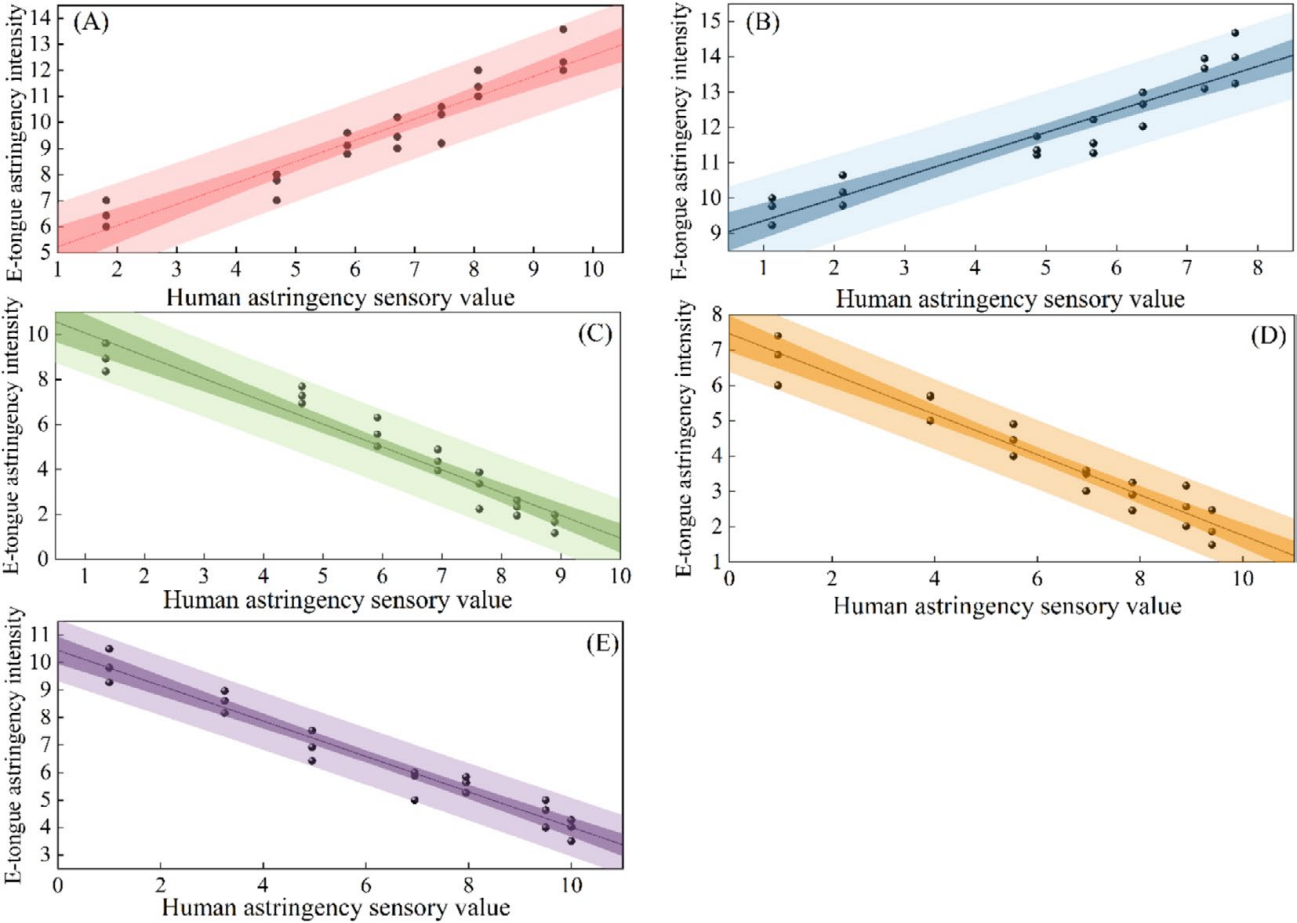
Fitting curve of E‐tongue and human sensory astringency intensity. Panels correspond to (A) epigallocatechin gallate (EGCG), (B) epigallocatechin (EGC), (C) tea polyphenols, (D) tannic acid, and (E) procyanidin. The shaded band around each regression line represents the 95% confidence interval, while the lighter outer band represents the 95% prediction interval.

Quantitative prediction models were developed for five responsive compounds (EGCG, EGC, tannic acid, tea polyphenols, and procyanidin) by correlating sensory intensity (*x*‐axis) with electronic tongue measurements (*y*‐axis), while chlorogenic acid and gallic acid were excluded due to their weak sensor responses. The modeling results are summarized in Figure 3 and Table 3.

**TABLE 3:** Regression model between E‐tongue response and human sensory for astringent compounds.

No.	Astringent compounds	Fitting curve	*R* ^2^	Coefficient (95% CI)
1	EGCG	y = 0.76*x* + 4.66	0.967	(0.67, 1.07)
2	EGC	y = 0.63*x* + 8.61	0.904	(0.57, 0.73)
3	Tea polyphenols	y = −0.98*x* + 10.96	0.945	(−1.15, −0.86)
4	Tannic acid	y = −0.5*x* + 6.74	0.920	(−0.64, −0.49)
5	Procyanidin	y = −0.63*x* + 10.41	0.988	(−0.71, −0.57)

Abbreviations: EGC, epigallocatechin; EGCG, epigallocatechin gallate.

The correlation patterns revealed distinct compound‐specific behaviors: EGCG and EGC showed positive linear relationships between instrumental and sensory measurements, where E‐tongue responses increased proportionally with perceived astringency; the calibration curve for EGCG exhibited a slope of 0.76, whereas that for EGC had a slope of 0.63; the 95% CI of the corresponding regression coefficients are 0.67–1.07 and 0.57–0.73, both of which are positive values (Figure 3, Table 3). Conversely, tea polyphenols, tannic acid, and procyanidin exhibited negative linear correlations, with instrument signals decreasing as sensory intensity increased; the respective slopes for these compounds were −0.98, −0.50, and −0.63; the 95% CI of the corresponding regression coefficients are −1.15 to 0.86, −0.64 to 0.49, and −0.71 to 0.57, all of which are negative values (Table 3). The variance in the slopes of the calibration curves reflects fundamental differences in the interaction modes between compounds of varying structure and the sensor membrane interface. The positive correlation of EGCG and EGC likely arises from their pyrogallol/galloyl groups (Xie et al. 2024). These moieties' strong hydrogen‐bond donating ability and hydrophobicity enable specific, directional interactions with the AE1 sensor membrane, generating a positive signal. Conversely, tea polyphenols, tannic acid, and procyanidin show a negative correlation, probably due to their higher molecular weight and phenolic hydroxyl count, leading to polymerization or complex structures (Rui et al. 2017). Their dominant interactions—nonspecific adsorption and physical shielding—may suppress the response or reverse the potential shift. Macromolecular aggregation on the membrane surface, hindering electroactive species diffusion, presents another plausible mechanism for signal attenuation. Notably, procyanidin displayed the strongest linear fit (*R* ^2^ = 0.988), followed by EGCG, while EGC showed a relatively weaker but still significant correlation (*R* ^2^ = 0.904) (Table 3). These robust correlations (all *R* ^2^ > 0.9) confirmed that the E‐tongue can effectively quantify and differentiate astringency patterns that align with human sensory perception (Xia et al. 2024). Furthermore, in all established regression models, the 95% confidence intervals for the regression coefficients of sensory intensity excluded zero, confirming their statistical significance. This confirms that, provided the model's assumptions are met, the effect of sensory intensity on the electronic tongue's astringency signal is statistically significant for all five compounds.

To further validate the model, sensory evaluations and electronic tongue tests were conducted for the astringent compounds at their validation concentrations. The experimental values were then statistically compared to the predicted values generated by the astringency model; the results are presented in Table 4.

**TABLE 4:** Validation of predictive models for the astringency intensity of astringent compounds.

No.	Astringent compounds	*R* ^2^	RMSRE%	MRE%
1	EGCG	0.971	2.400	2.526
2	EGC	0.920	4.813	2.866
3	Tea polyphenols	0.959	7.149	5.112
4	Tannic acid	0.967	5.076	3.856
5	Procyanidin	0.956	2.212	2.197

Abbreviations: EGC, epigallocatechin; EGCG, epigallocatechin gallate.

As shown in Table 4, the coefficients of determination (*R* ^2^) for the five astringency calibration models ranged from 0.920 to 0.971, all exceeding the threshold of 0.9. This indicates a strong correlation between the sensorially measured values and the instrumentally predicted values, confirming the models' accuracy in capturing the underlying relationship. The models' predictive performance was further assessed using the root mean square relative error (RMSRE) and mean relative error (MRE). For all five models, the RMSRE and MRE values ranged from 2.197% to 7.149%, well below the 10% benchmark (Table 4). These low error rates affirm that the validation models are highly accurate and possess excellent predictive capability.

### 3.4 Difference Analysis of Electronic Tongue Taste Indices Across Varying Concentrations of Astringent Compounds

**FIGURE 4:**
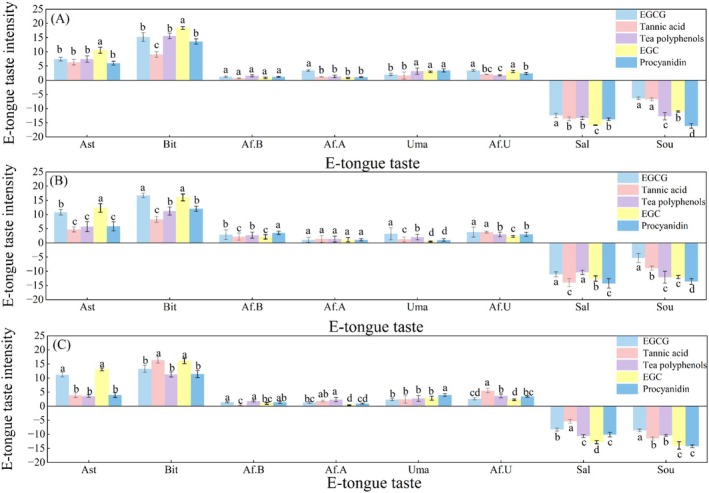
Different analysis of taste profile differences for astringent compounds at various concentrations using an E‐tongue. (A) 0.5 g/L, (B) 1.0 g/L, (C) 1.5 g/L. EGC, epigallocatechin; EGCG, epigallocatechin gallate. Different letters indicate significant differences at the *p* < 0.05 level (ANOVA with Duncan test).

To systematically evaluate the overall taste characteristics and differences among various astringent compounds, E‐tongue technology was employed to analyze the taste properties of five compounds—EGCG, EGC, tannic acid, tea polyphenols, and procyanidin—at concentrations of 0.5, 1.0, and 1.5 g/L (Figure 4). The E‐tongue results revealed that each compound exhibited a unique and complex taste profile, with significantly distinct response patterns across the different sensors.

All tested compounds elicited the strongest positive signals from the astringency and bitterness sensors (Figure 4). Among them, epigallocatechin (EGC) exhibited a significantly higher astringency response value at every concentration (10.54 ± 1.05 at 0.5 g/L; 12.29 ± 1.51 at 1.0 g/L; 13.07 ± 0.52 at 1.5 g/L) compared to all other compounds (*p* < 0.05). At the highest concentration of 1.5 g/L, the astringency intensity of EGC (13.07 ± 0.52) was significantly higher than that of EGCG (11.19 ± 0.78), procyanidin (3.98 ± 0.84), tannic acid (3.90 ± 0.78), and tea polyphenols (3.59 ± 0.44). Furthermore, the astringency intensities of tannic acid, procyanidin, and tea polyphenols belonged to the same statistical significance group (*p* > 0.05), indicating that their astringency levels could not be distinguished statistically.

Conversely, the response values for all compounds on the umami, bitter aftertaste, umami aftertaste, and astringent aftertaste sensors were negligible and close to zero (Figure 4). No significant differences were found between different compounds or concentrations (*p* > 0.05) for these taste attributes. This indicates that the compounds themselves do not impart a primary umami taste, nor do their bitterness and astringency characteristics exhibit persistence, as no significant aftertaste was detected. This result also confirms the E‐tongue's capability to effectively discriminate between primary taste and aftertaste signals.

Notably, all compounds produced significant negative responses on the salty and sour sensors (Figure 4). We speculate that this phenomenon may occur because the phenolic hydroxyl groups in the polyphenolic compounds strongly bind to or adsorb on specific sites of the sensor membranes. This interaction could mask the membrane's normal response to salty and sour stimuli, leading to a pronounced decrease in the signal current relative to the reference solution and thus resulting in a negative value.

In conclusion, the E‐tongue effectively distinguishes the primary taste attributes of astringent compounds and provides a valuable tool for analyzing their underlying taste mechanisms.

### 3.5 Principal Component and Cluster Analysis

**FIGURE 5:**
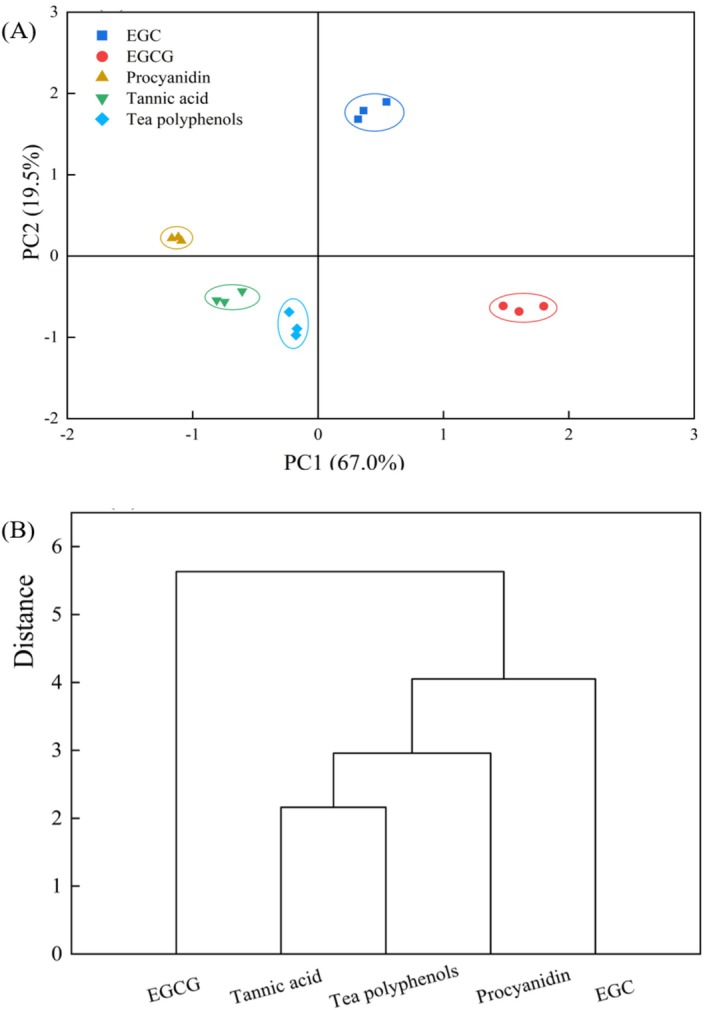
Principal component analysis (PCA) score plot (A) and cluster analysis dendrogram (B) of the five astringent compounds. EGC, epigallocatechin; EGCG, epigallocatechin gallate.

To evaluate the discrimination capability of the E‐tongue for five astringent compounds (EGCG, EGC, tannic acid, tea polyphenols, and procyanidin), principal component analysis (PCA) and cluster analysis were employed (Figure 5).

The PCA score plot shows that PC1 and PC2 accounted for 67.0% and 19.5% of the total variance, respectively, with a cumulative contribution rate of 86.5%, indicating that these two components sufficiently captured the majority of sample characteristics (Figure 5A). Notably, EGC in the positive of PC1 and PC2, procyanidin in the negative of PC1 and positive of PC2, tannic acid and tea polyphenols clustered in the negative of PC1 and PC2, and EGCG in the positive of PC1 and negative of PC2 (Figure 5A). This clear spatial separation demonstrates that PCA effectively distinguishes among the five astringent compounds (EGCG, tannic acid, tea polyphenols, EGC, and procyanidin) detected by the E‐tongue. Hierarchical cluster analysis was applied to the taste indicators of the astringent compounds, with the results presented in Figure 5B. At an intercluster distance threshold of 2.5, the compounds separated into four clusters. EGCG, EGC, and procyanidin each formed distinct clusters, consistent with their separate positions in the PCA score plot. Meanwhile, tannic acid clustered with tea polyphenols, aligning with their colocation in the negative quadrant of the PCA plot. These cluster analysis results are in full agreement with the PCA pattern.

Based on the combined results of principal component analysis (PCA) and cluster analysis, the five astringent compounds were categorized into four distinct groups: Group 1 (EGCG), Group 2 (EGC), Group 3 (procyanidin), and Group 4 (tannic acid and tea polyphenols). This classification confirms that these compounds can be systematically grouped according to their shared taste characteristics. Our findings are consistent with those reported by Zhang et al. (2022), who similarly employed PCA and cluster analysis for effective category classification of astringent compounds. This methodological alignment further validates our analytical approach.

## 4 Conclusion

In this study, the E‐tongue analysis effectively characterized five astringent compounds (EGCG, EGC, tannic acid, tea polyphenols, and procyanidin), whose astringency intensity was positively correlated with concentration in a logarithmic manner. In contrast, gallic acid and chlorogenic acid showed negligible responses and were excluded from the model. Taste quality analysis revealed that EGCG, EGC, tannic acid, tea polyphenols, and procyanidin showed logarithmically concentration‐dependent astringency and dominant bitterness/astringency responses without umami or aftertaste. Furthermore, principal component analysis (PCA) and cluster analysis confirmed the E‐tongue's ability to effectively discriminate among the five astringent compounds. Regression analysis between E‐tongue measurements and human sensory scores demonstrated strong correlations for EGCG, EGC, tea polyphenols, tannic acid, and procyanidin, with procyanidin and EGCG exhibiting the best linearity. Five quantitative prediction models established based on these correlations showed high predictive accuracy in the validation set (correlation coefficient > 0.9; RMSE < 10%), validating the E‐tongue as a robust tool for rapid quality assessment and product development of astringent compounds.

## Author Contributions

**Meng‐Yao Wang:** investigation (equal), methodology (equal), visualization (equal), writing – original draft (equal). **Zhao‐Lin Sun:** conceptualization (equal), data curation (equal), resources (equal). **Juan Lü:** formal analysis (equal), software (equal), supervision (equal). **Hao Zhu:** resources (equal), software (equal), supervision (equal). **Zeng‐Hui Zhang:** funding acquisition (equal), supervision (equal), validation (equal). **Xi Zhang:** data curation (equal), formal analysis (equal), investigation (equal). **Zhi‐Gang Guo:** conceptualization (equal), supervision (equal). **Yao Wang:** funding acquisition (equal), validation (equal), visualization (equal). **Jing Yang:** funding acquisition (equal), project administration (equal), writing – review and editing (equal).

## Funding

The work was supported by the Henan Province Science and Technology Research Project (252102320216).

## Ethics Statement

This study involved sensory analyses conducted by a trained panel, following the ethical guidelines of the 1964 Helsinki Declaration and its later amendments, and informed consent was obtained from each subject before they participated in the study. This information sheet describes the study procedures, potential risks, and participants' rights. Participants were explicitly informed of their right to decline participation in the study or to withdraw their consent at any stage without facing any negative consequences. While there was no formal ethics committee available, the research followed the principles and guidelines outlined in the Helsinki Declaration to ensure the ethical treatment of participants.

## Consent

Written informed consent was obtained from all study participants.

## Conflicts of Interest

The authors declare no conflicts of interest.

## Data Availability

Research data are not shared.
